# Therapeutic Endoscopy in Combination with Quadruple Therapy in Treating Bleeding Caused by Gastric Ulcer

**DOI:** 10.12669/pjms.341.13418

**Published:** 2018

**Authors:** Yuqin Zuo, Shuhui Wang, Xiujuan Cui, Hongna Lv

**Affiliations:** 1 Yuqin Zuo, Department of Gastroenterology, Binzhou People's Hospital, Shandong, 256600, China; 2 Shuhui Wang, Department of Ophthalmology, Binzhou People's Hospital, Shandong, 256600, China; 3 Xiujuan Cui, Department of Neurosurgery, Binzhou People's Hospital, Shandong, 256600, China; 4 Hongna Lv, Department of Gastroenterology, Binzhou People's Hospital, Shandong, 256600, China

**Keywords:** Bleeding gastric ulcer, Therapeutic endoscopy, Quadruple therapy

## Abstract

**Objective:**

To analyze the efficacy of therapeutic endoscopy in combination with quadruple therapy in treating bleeding caused by gastric ulcer and investigate the factors inducing rebleeding.

**Methods:**

Two hundred and twelve patients with bleeding caused by gastric ulcer who were admitted to Binzhou People's Hospital, Shandong, China between April 2015 and April 2016 were selected as research subjects. The patients were randomly divided into a control group and an experimental group. Patients in the control group were treated by quadruple therapy, while patients in the observation group received therapeutic endoscopy treatment in addition to the same treatment as the control group. The treatment efficacy, adverse reaction, H pylori (Hp) clearance rate and rebleeding were compared between the two groups.

**Results:**

The effective rate of the observation group was 98.1%, which was significantly higher than that of the control group (80.2%), and the difference had statistical significance (P<0.05). The incidence of adverse reactions in the observation group was lower than that in the control group. The Hp clearance rate of the observation group was higher than that of the control group, and the difference had statistical significance (P<0.05). The multi-factor analysis on rebleeding suggested that whether therapeutic endoscopy was performed or not, hemoglobin level and presence of peptic ulcer stage A1 were independent risk factors.

**Conclusion:**

Endoscopic treatment in combination with quadruple therapy is better in the treatment of bleeding caused by gastric ulcer as compared to medical treatment alone. Patients with high-risk factors such as low content of hemoglobin and ulcer at stage A1 should be monitored more carefully to prevent the occurrence of rebleeding.

## INTRODUCTION

Gastric ulcer is one of the commonly seen digestive system diseases in clinics. With the improvement of living standards and changes in dietary habits, the incidence of gastric ulcer is increasing.^1^,^2^ Singh et al has suggested that gastric ulcer is responsible for 35% of cases with upper digestive tract bleeding.^3^ Gastric ulcer induced bleeding is the most common complication for patients with gastric ulcer and also one of medical emergency. Rebleeding in patients with gastric ulcer bleeding can decrease the survival rate of patients.^4^ If treatment is delayed, the life of patients will be severely threatened. Therefore, it is important to select a scientific treatment for patients with gastric ulcer induced bleeding.

The pathogenesis of gastric ulcer has not been studied thoroughly; however, weakening of protection effect caused by Helicobacter pylori (HP) infection and excessive secretion of gastric acid has been agreed.^5^,^6^ Treatment with drugs such as acid inhibitors (prilosec and lansoprazole), anti-inflammatory drugs (levofloxacin and amoxicillin) and gastric mucosal protective agents (bismuth potassium citrate) were the main therapy for gastric ulcer induced bleeding previously. Though drug treatment has certain efficacy, it fails to control rebleeding.

In recent years, the efficacy of endoscopy in the treatment of gastric ulcer induced bleeding has been extensively recognized. Using therapeutic endoscopy can identify the cause of bleeding; then surgery can be performed to stop bleeding if needed.^8^ Few studies focus on the treatment of gastric ulcer with therapeutic endoscopy in combination with quadruple therapy. This study investigated the efficacy of therapeutic endoscopy in combination with quadruple therapy in the treatment of bleeding gastric ulcer and compared it to medical treatment alone.

## METHODS

Two hundred and twelve patients with gastric ulcer induced bleeding who were admitted to the hospital between April 2015 and April 2016 were selected as research subjects. The symptoms of the patients satisfied the diagnostic criteria of gastric ulcer induced bleeding.^9^ Major symptoms of the patients included haematemesis, acid regurgitation and dizziness. All the patients were diagnosed to have gastric ulcer by gastroscopy and had positive histopathological examination result of HP. Those who had mental diseases, disturbance of consciousness, liver and kidney diseases or other digestive diseases or had contraindication to the drugs used in the present study and pregnant women were excluded. The patients were randomly divided into a control group and an observation group, 106 each. The differences of general data between the two groups had no statistical significance (P>0.05) (Table-I). This study was approved by the ethics committee of the hospital and all the patients signed informed consent.

**Table-I:** Comparison of general data between the two groups.

Group	Gender (male/female)	Age (years)	Course of disease (year)	Forrest grading				

				*Ia*	*Ib*	*Ic*	*IIa*	*IIb*
Observation group	60/46	2.43±6.14	5.12±2.24	39	25	25	12	6
Control group	58/48	53.12±5.73	5.01±1.98	38	27	23	14	4
t/X^2^	0.334	0.438	0.512	0.183	0.209	0.174	0.225	0.197
P	>0.05	>0.05	>0.05	>0.05	>0.05	>0.05	>0.05	>0.05

### Treatment Methods: Therapy in the control group

Patients in the control group were treated by quadruple therapy, i.e., 1000 mg of amoxicillin (Guangzhou Baiyunshan Pharmaceutical Factory of Guangzhou Baiyunshan Pharmaceutical Co., Ltd., Guangzhou, China; batch. No.: H44021518), 2000 mg of levofloxacin (Nanjing Zhengke Pharmaceutical Co., Ltd., Guangzhou, China; batch. No.: H20074085), lansoprazole (Jiangsu Kangyuan Pharmaceutical Inc., China; Batch No.: H20067606) and 220 mg of bismuth potassium citrate (Lizhu Pharmaceutical Factory of Lizhu Group; batch No.: H10900084), twice each day. The treatment lasted for seven days. If rebleeding did not occur, patients could take liquid diet one day after treatment, semi-liquid diet two days after treatment and full diet seven days after treatment.

### Therapy in the observation group

Under the assistance of therapeutic endoscopy, the bleeding area was washed by normal saline, blood crust was treated, and the bleeding points were checked. Then ring loop ligature was used to stop bleeding. The patients were forbidden to eat for one day after thorough hemostasis. Quadruple therapy was used on the second day after surgery. The process and dosage were the same with the control group. If there was no rehaemorrhagia, they were allowed to eat liquid food one day after surgery, semi-liquid food two days after surgery and normal food seven days after seven days.

### Clinical observation indicators

The clinical treatment efficacy was compared between the two groups. The criteria for successful outcome were as follows.^10^ Treatment was considered successful if bleeding absolutely stopped within 72 hour after treatment, gastric ulcer completely disappeared, or scar appeared. Treatment was considered as effective if bleeding was relieved 72h after treatment and area of gastric ulcer narrowed for no less than 50%. Treatment was determined as ineffective if bleeding did not stop 72 hour after treatment and area of gastric ulcer narrowed for less than 50%. The calculation formula for overall effective rate was overall effective rate=effective rate + significantly effective rate.

The incidences of adverse reactions including dizziness, nausea and fever and rehaemorrhagia were compared between the two groups. The Hp clearance rate of the two groups was also compared. Negative result in HP rapid urase test after treatment indicated absolute clearance. In addition, the condition of rebleeding of the two groups was compared and analyzed. Rebleeding within two weeks after treatment and amount of bleeding exceeded 1000 mL in two days were defined as rebleeding. Once there was rebleeding, the patients were transferred to department of surgery for treatment. The indication for bleeding control was no haematemesis and black stool and absence of progressive hemorrhage after treatment.

### Statistical method

Data were statistically analyzed by SPSS ver. 19.0. Measurement data were expressed as mean±standard deviation (SD) and processed by t test. Enumeration data were expressed as case (%) and processed by Chi-square test. Multi-factor analysis was performed using Logistic regression. Difference was considered as statistically significant if P<0.05.

## RESULTS

**Fig. 1:**
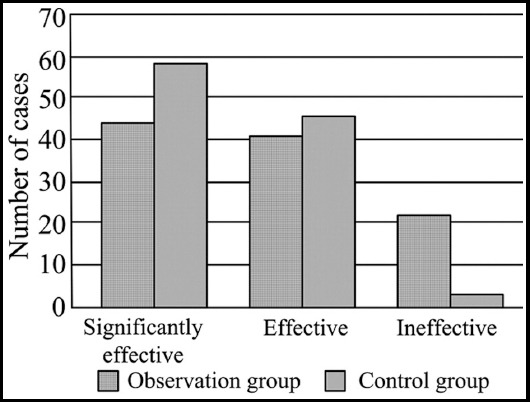
Comparison of clinical treatment efficacy between the two groups.

The overall efficacy in the observation group was 98.1%, which was higher than that of the control group (80.2%). The difference of data between the two groups had statistical significance (X^2^=12.371, P<0.05; Fig.1).

### Incidence of adverse reactions

The incidence of adverse reactions of the observation group was 4.7%; there were two cases of dizziness, two cases of nausea and one case of fever. The incidence of adverse reactions was 21.7%; there were 12 cases of dizziness, seven cases of nausea and four cases of fever. The incidence of adverse reactions of the observation group was lower than that of the control group, and the difference had statistical significance (X^2^=6.131, P<0.05).

### Rehaemorrhagia

The incidence of rehaemorrhagia of the observation group was 8.5% (9/106), which was lower than 30.2% in the control group (32/106). The difference between the two groups was statistically significant (X^2^=7.103, p<0.05).

### Single-factor and multi-factor analysis on factors affecting rebleeding

To analyze the risks factors associated with rebleeding, two hundred and twelve patients were divided into a rebleeding group and a non-rebleeding group. Different indicators were analyzed. Among the single factors affecting rebleeding, the difference of age and gender ratio had no statistical significance; the difference of ulcer at stage A1, amount of bleeding, content of blood platelet and hemoglobin and endoscopic treatment or not had statistical significance (P<0.05, Table-II). The Logistic regression analysis on multiple single factors suggested that level of hemoglobin, ulcer at stage A1 and endoscopic treatment or not were independent risk factors for rebleeding (Table-III).

**Table-II:** The single-factor analysis on rebleeding.

Influence factors	Non-bleeding group	Bleeding group	t/X2	P
Gender (male/female)	94/77	24/17	0.527	>0.05
Age (years)	55.46±6.2	54.97±6.3	0.633	>0.05
Ulcer at stage A1 (%)	15(36.6%)	10(5.8%)	9.728	<0.05
Amount of bleeding (mL)	141.93±10.17	236.79±13.58	14.571	<0.05
Hemoglobin (g/L)	108.35±12.33	84.36±6.22	12.135	<0.05
Blood platelet (×10^9^/L)	185.44±12.12	123.76±11.13	10.362	<0.05
Undergoing endoscopic treatment (%)	97(56.7%)	9(22.0%)	14.689	<0.05

**Table-III:** The multi-factor analysis on bleeding.

Factor	Mean±SD	P	OR	95%CI
Ulcer at stage A1	1.27±0.41	0.026	1.947	1.045-3.123
Hemoglobin	1.33±0.24	0.013	2.875	1.127-4.158
Endoscopic treatment or not	1.83±0.42	0.021	2.683	1.441-6.862

OR: odd ratio; CI: confidence interval.

## DISCUSSION

Gastric ulcer induced bleeding is affected by multiple complex factors including gastric mucosal injury caused by increased gastric acid and pepsin, weakening of protective effect of mucus and mucous membrane and HP infection. Therefore, it is necessary to focus on inhibiting secretion of gastric acid, protecting gastric mucosa and clearing HP infection.^11^ Quadruple therapy is the most frequently used therapy in previous clinical treatment. It can effectively relieve symptoms of gastric ulcer and avoid other complications.^12^ Lansoprazole, a high-efficient anti-acid drug is beneficial to the inhibition of gastric acid.^13^ Levofloxacin and amoxicillin are indispensable antibiotics in quadruple therapy, which can promote the rapid healing of gastric ulcer.^14^ Bismuth potassium citrate, a gastric mucosal protective agent, can avoid the invasion of gastric acid to ulcer and reduce the incidence of complications.^15^,^16^ Quadruple therapy has favorable efficacy; however, drug treatment only is difficult to rapidly relieve bleeding symptom and the rehabilitation of patients may also be affected. Therefore, patients should undergo other treatment in addition to quadruple therapy to ensure treatment efficacy.

With the rapid development and extensive application of endoscopic technique, the efficacy of therapeutic endoscopy in the diagnosis and treatment of gastric ulcer has been constantly highlighted. Therapeutic endoscopy can help find out pathogenesis in short term and bleeding spots; as a result, doctors can timely stop bleeding under therapeutic endoscopy to reduce frequency of hemostasis, relieve pain of patients and prevent and reduce the occurrence of adverse events. Yuan W et al. found that the hemostasis effective rate of therapeutic endoscopy in combination with quadruple therapy was 96.2% and the rebleeding incidence was 15.8%.^17^ This study suggested that the overall effective rate of the observation group was 98.1%, which was higher than that of the control group (80.2%); the incidence of rebleeding of the observation group was 8.5%, which was significantly lower than that of the control group (30.2%) (P<0.05), which was consistent with relevant literature.^18^

Moreover, the risk factors of rebleeding were analyzed. It was found that the secondary bleeding was more serious than the primary bleeding; patients who had a large amount of bleeding, ulcer at stage Ai, low content of hemoglobin and blood platelet and did not undergo endoscopic treatment had high risks of bleeding. Multi-factor regression analysis suggested that endoscopic treatment or not, content of hemoglobin and ulcer at stage A1 were independent risk factors, which were consistent with the literature.^19^,^20^

## CONCLUSION

Therapeutic endoscopy in combination with quadruple therapy can improve the treatment efficacy and effectively reduce the incidences of adverse reactions and rebleeding. The combined therapy is worth promotion in clinical practice. Endoscopic treatment or not, content of hemoglobin and ulcer at stage A1 are risk factors for rebleeding. Patients with the aforementioned clinical characteristics should be closely observed; surgical treatment should be performed if necessary.

### Authors' Contribution

**YQZ & SHW:** Study design, data collection and analysis.

**YQZ, SHW & XJC:** Manuscript preparation, drafting and revising.

**YQZ & HNL:** Review and final approval of manuscript.
